# Ex-Vivo Adhesion of *Enterococcus faecalis* and *Enterococcus faecium* to the Intestinal Mucosa of Healthy Beagles

**DOI:** 10.3390/ani11113283

**Published:** 2021-11-16

**Authors:** Mohsen Hanifeh, Thomas Spillmann, Mirja Huhtinen, Yannes S. Sclivagnotis, Thomas Grönthal, Ulla Hynönen

**Affiliations:** 1 Department of Equine and Small Animal Medicine, Faculty of Veterinary Medicine, University of Helsinki, 00014 Helsinki, Finland; thomas.spillmann@helsinki.fi (T.S.); thomas.gronthal@helsinki.fi (T.G.); 2 Orion Corporation, Orion Pharma, R&D, 02200 Espoo, Finland; mirja.huhtinen@orionpharma.com (M.H.); Yannes.SclivagnotisSiotkas@orion.fi (Y.S.S.); 3 Department of Veterinary Biosciences, Veterinary Microbiology and Epidemiology, University of Helsinki, 00014 Helsinki, Finland; ulla.hynonen@helsinki.fi

**Keywords:** bacterial adhesion, *Enterococcus faecalis*, *Enterococcus faecium*, dogs, chicken, mucosa

## Abstract

**Simple Summary:**

Bacterial adhesion to the intestinal mucosa appears to be an important feature for probiotics. When selecting bacteria for probiotic use, those with high ability to attach to the intestines are preferred. Specific strains of *Enterococcus faecalis* and *Enterococcus faecium* have been used as probiotics or feed additives. Due to the lack of information in dogs, we aimed (1) to investigate the intra- and inter-host species adhesion of *E. faecalis* and *E. faecium* to the duodenal mucosa of six healthy beagles using bacteria derived from dogs and chickens, and (2) to validate a method of quantifying the adhesion of Alexa Fluor stain-labeled bacteria to paraffin-embedded canine duodenal mucosa. Our results show that both canine- and chicken-derived *E. faecalis* strains adhered better than *E. faecium* to the duodenal mucosa of beagles. In addition, canine *E. faecalis* and *E. faecium* adhere in higher numbers to canine duodenal mucosa, compared to chicken-derived strains of the same species. Our results suggest that both the bacterial strain and the host species may influence the mucosal adhesion properties of *E. faecalis* and *E. faecium*.

**Abstract:**

Some *Enterococcus faecalis* and *E. faecium* strains are used as probiotics or feed additives. Adherence to the intestinal mucosa is considered a crucial step for intestinal bacteria to colonize and further interact with the host epithelium and the immune system. In dogs, there are no studies investigating the adhesion of *E. faecalis* and *E. faecium* to paraffin-embedded intestinal mucosa. Therefore, we aimed to investigate the adhesion of *E. faecalis* and *E. faecium* to the intestinal mucosa of six healthy beagles using bacteria derived from dogs and chickens. In addition, we aimed to validate a method to test the adhesion of Alexa Fluor-labeled bacteria to paraffin-embedded canine intestinal mucosa. The results of our study show that both canine- and chicken-derived *E. faecalis* strains adhered significantly better than *E. faecium* to the duodenal mucosa of healthy beagles (*p* = 0.002). In addition, canine *E. faecalis* and *E. faecium* adhered in higher numbers to canine duodenal mucosa, compared to chicken-derived strains of the same species (*p* = 0.015 for *E. faecalis* and *p* = 0.002 for *E. faecium*). The determination of the hydrophobicity of bacteria revealed that canine *E. faecalis* had the highest hydrophobicity level (36.6%), followed by chicken *E. faecalis* (20.4%), while canine *E. faecium* (5.7%) and chicken *E. faecium* (4.5%) had the lowest levels. Our results suggest that both the bacterial species and the host origin of the strain may influence mucosal adhesion.

## 1. Introduction

Many commensal and pathogenic bacteria in the gastrointestinal tracts of humans and animals express various adhesin molecules (e.g., FirmH, FadA, Ace), allowing them to bind to various surfaces in the host [1,2,3]. Bacterial adherence to the intestinal epithelium and mucus is considered a crucial step for intestinal bacteria to colonize and further interact with the host epithelium and the immune system [4,5]. Therefore, bacterial adhesion to the intestinal mucosa is considered an important feature for probiotics, and microorganisms with a high ability to attach to the intestines are preferred when selecting microorganisms for probiotic use [6,7].

Probiotics can enhance mucosal health by several proposed mechanisms, including the production of short-chain fatty acids (SCFAs), antimicrobial substances, modulation of the immune response, competitive exclusion of pathogenic bacteria through interference with their adherence to the intestinal mucosa, and enhancement of epithelial barrier functions [5,8]. There has been controversy about the host-specificity of probiotic bacteria. On the one hand, Rinkinen et al. (2003b) concluded that the intestinal mucus adhesion properties of lactic acid bacteria are more dependent on the strain than on the host. On the other hand, the probiotic characteristics of microorganisms are reportedly related to host specificity; therefore, for successful use as a probiotic, the bacterial species should be of host intestinal origin [9,10]. Most probiotics currently available for dogs are not originally derived from the canine gut microbiota [11].

The fecal microbiome of healthy dogs is co-dominated by three phyla: *Fusobacteria, Bacteroidetes,* and *Firmicutes* [12]. Within this core bacterial community, *Enterococcus* spp., as normal inhabitants of the gut, are widely studied as potential candidate probiotics. They belong to lactic acid bacteria of the phylum *Firmicutes* and have the functional requirements of probiotics, which include tolerance to gastric juice and bile, adherence to intestinal epithelial surfaces, modulation of the immune response, antagonistic activity towards intestinal pathogens by producing bacteriocins, and the capacity to stabilize and modulate the intestinal microbiota [13]. While the genus *Enterococcus* includes many species, only a few have been studied for probiotic use, such as *E. faecalis and E. faecium* [14]. In humans, these are frequently used as probiotics to promote human health or to treat diseases/disorders such as diarrhea, irritable bowel syndrome, or antibiotic-associated diarrhea [15]. In dogs and cats, some studies have shown the beneficial effects of *E. faecium* in treating diarrhea [16,17]; however, others did not find significant effects of using *E. faecium* for treating dogs with food-responsive diarrhea, kennel stress-associated diarrhea, or giardiasis [8]. Cerquetella et al. (2012) reported the use of various inactivated bacteria, including *E. faecalis,* to successfully reduce the number and severity of diarrhea episodes in five out of six dogs with recurrent episodes of self-limiting diarrhea [18].

Many studies investigating host–microbiome interactions have used cell lines, tissue culture, or immobilized intestinal mucus to investigate bacterial adhesion [5,19,20,21,22]. However, tissue sections (e.g., paraffin-embedded tissue samples) offer a more physiological context to the adhesion study, as they provide cellular organization and structures that are nearly impossible to obtain using in vitro cell culture [23]. Therefore, bacterial adhesion to duodenal tissue sections was used in this study to investigate the microanatomic context of bacterial adherence. Kainulainen et al. (2015) investigated the adhesion of lactic acid bacteria to canine intestinal epithelium, and revealed not only adherence, but also intestinal barrier fortifying and anti-inflammatory effects [5]. To our knowledge, there are no studies on dogs investigating the adhesion of *E. faecalis* and *E. faecium* to paraffin-embedded intestinal mucosa.

The primary aim of our current study was to investigate the adhesion capacities of *E. faecalis* and *E. faecium* to the intestinal mucosa of healthy dogs using strains derived from dogs, for intra-host species comparison, and from chickens, for inter-host species comparison. As there is a relationship between the degree of bacterial adhesion and hydrophobicity, we also performed hydrophobicity testing with the investigated strains [24]. In addition, we aimed to validate a method to test bacterial adhesion on paraffin-embedded canine intestinal mucosa. 

## 2. Materials and Methods

### 2.1. Bacteria and Growth Conditions

The bacteria used in this study are listed in Table 1. Canine *E. faecalis* was isolated from the feces of a healthy Cocker Spaniel and canine *E. faecium* from the feces of a healthy Sheltie–Rough Collie mixed dog. Chicken *E. faecalis* and *E. faecium* were isolated from the cecum of one healthy broiler hen. They were first cultured in blood agar medium (Thermo Scientific ™ PB5012A) overnight at +37 °C, after which the bacterial colonies were inoculated into brain heart infusion (BHI) broth (Oxoid Ltd, Basingstoke, Hampshire, UK) and cultivated overnight at +37 °C.

### 2.2. Duodenal Tissue Samples

Duodenal paraffin-embedded tissue samples from six healthy laboratory beagle dogs were used. All beagles were intact females of the same age (6 years old) and with a median body weight of 12.5 kg (range 10.2–14.2 kg). Duodenal samples of healthy beagles were collected during post-mortem examinations at the end of an unrelated study (ethical license ESAVI/7290/04.10.03/2012) approved by the Finnish National Animal Experiment Board. All beagles were considered healthy based on history, physical examination, complete blood count, serum biochemistry profile, fecal examination, and histological evaluation. All the duodenal tissue samples were fixed in 4% formaldehyde in phosphate-buffered saline (PBS), embedded in paraffin, and sectioned to the thickness of 3–5 µm. Three sections from each duodenal tissue sample were mounted to each studied slide.

### 2.3. Dye Preparation

Alexa Fluor 488 NHS Ester (Thermo Fisher Scientific, Rockford, IL, USA; Cat. no.: A20000) was used as a staining dye. To prepare dry aliquots of Alexa Fluor 488 (AF488), 1 mg of lyophilized AF488 was dissolved in 2 mL of ultrapure methanol to achieve a final concentration of 0.5 mg/mL. Then, the solution was divided in aliquots in Eppendorf tubes with different quantities of AF488 per tube. The tubes were kept at −80 °C for at least 30 min, and the solvents were vaporized in a vacuum centrifuge (DNA mini), protected from light. All dried aliquots of AF488 were stored protected from light and moisture at −20 °C until further analysis.

### 2.4. Collecting and Labeling Bacteria

*E. faecalis* and *E. faecium* cultured in broth were collected by centrifugation at 4500× *g* for 10 min at +4 °C. The supernatants were removed by pipetting, and the cells were washed twice by adding 0.1 M NaHCO_3_ (pH 8.3) and centrifuging again. The collected cells were finally re-suspended in 1.5 mL of 0.1 M NaHCO_3,_ their OD_600_ values were measured, and the bacterial suspensions were adjusted to the OD_600_ value of 1.0 with 0.1 M NaHCO_3_.

In total, 0.5 mL aliquots of the bacterial suspensions were added to tubes containing 20 µg of AF488 and mixed well by pipetting. The tubes were wrapped in foil and incubated for 1 h at room temperature (RT) with end-over-end rotation. The stained cells were then centrifuged at 16,000× *g* for 5 min at +4 °C, the supernatants were removed, and the cells were washed thrice with 12.5 mM Tris (pH 7.4) and 80 mM NaCl (tris-buffered saline, TBS) protected from light. To minimize the nonspecific binding of bacterial cells to the duodenal epithelium, the harvested cells were re-suspended in 0.5 mL of blocking solution (2% bovine serum albumin, 12% fetal bovine serum, 0.2% Triton X-100 in TBS) containing 0.001% Tween 20 and kept protected from light and on ice. The labeling of bacterial cells was checked using a Leica DM 4000B epifluorescence microscope (Leica Microsystems, Wetzlar, Germany). OD_600_ values of the labeled bacterial suspensions were checked, re-adjusted to OD_600_ = 1, and diluted in blocking solution.

### 2.5. Bacterial Adhesion to Duodenal Mucosa

The adhesion assay was performed as described previously (Isaacson et al. 2018), with slight modifications. The paraffin-embedded duodenal tissue slides were first deparaffinized in xylene and ethanol. After deparaffinization, blocking solution was added and the slides were incubated for 6 h at RT. The blocking solutions were discarded and 10 µL measures of the labeled bacterial suspensions (OD_600_ = 1 and OD_600_ = 0.5) were gently placed on slides. The slides were incubated overnight at +4 °C in a moist chamber protected from light to allow the bacteria to bind. An overnight incubation temperature of +4 °C was chosen to protect the biological material from deterioration, as the incubation time was long.

The next day, the tissue slides were washed thrice in 200 mL PBS and stained with nucleic acid-binding dye 4,6′-diamidino-2-phenylindole (DAPI) (1 µg/mL in PBS). The slides were incubated for 3–15 min at RT protected from light and then washed three times with PBS. The slides were kept in the last washing solution and mounted one by one. To dry the mounting medium, the slides were kept at RT for 4 h protected from light. The adherent bacteria were examined by epifluorescence microscopy using an epifluorescence I3 filter to detect mucosa-attached fluorescent bacteria and a DAPI filter for identification of host cell nuclei. In addition, Epifluorescence I3 and a phase contrast combination filter were used to display the structure of the duodenal mucosa and its attached fluorescent bacteria at the same time. The captured images were digitally recorded using Cell^P imaging software (Olympus Corp., Tokyo, Japan). The mean number of stained bacteria attached to the mucosa was counted in 20 randomly selected fields (3.5 × 10^4^ μm^2^ each). Three replicates of the experiment were used to estimate the adhesion of each strain, and the results were reported as a mean of the three runs.

To control for unspecific binding, 20 µL of blocking solution was added to each well of a diagnostic slide (Waldemar Knittel Glasbearbeitungs GmbH, Braunschweig, Germany). The diagnostic slide was kept in a moist chamber at RT overnight. The next day, the diagnostic slides were washed once with TBS. Concurrently, while adding stained bacterial suspensions to tissue slides, 10 µL of each bacterial suspension was added to two parallel wells of the diagnostic slides, and from then on, these slides were treated identically to the tissue slides.

### 2.6. Measurement of Bacterial Hydrophobicity

The hydrophobicity of the investigated bacteria was determined using an in vitro method to detect the bacterial adhesion to hydrocarbons [25,26]. Canine and chicken *E. faecalis* and *E. faecium* were grown in BHI broth at +37 °C for 18 h. The bacterial cells harvested by centrifugation (5000× *g*, +4 °C, 15 min) were washed twice with PBS (pH7.0) and resuspended in the same solution to an optical density (OD600) of 0.552–0.606 (A0). In total, 1 mL of xylene (Bio-Optica, Milan, Italy) was added to 3 mL of cell suspension in a glass tube and vortexed for 2 min after 10 min of incubation at RT. After phase separation, the aqueous phase was removed after 2 h of incubation at RT, and the OD600 nm was determined (A1) and compared with the initial value. Due to the destructive effect of xylene on plastic cuvettes, both optical density measurements (A0 and A1) were performed using glass cuvettes. The percentage of hydrophobicity (%H) was calculated using the equation %H = [(A0 − A1)/A0] × 100, and was expressed as follows: 0–35%, low hydrophobicity; 36–70%, medium hydrophobicity; and 71–100%, high hydrophobicity [27]. All measurements were made in triplicate and the mean hydrophobicity percentage was reported.

### 2.7. Statistical Analyses

The normality of data distribution was checked with the Shapiro–Wilk test. The data were found to be non–normally distributed; therefore, nonparametric tests were performed. The differences between the numbers of canine *E. faecalis* (OD 0.5 and 1) and *E. faecium* (OD 0.5 and 1) cells and between chicken *E. faecalis* (OD 0.5 and 1) and *E. faecium* (OD 0.5 and 1) cells that adhered to the canine duodenal mucosa were determined using the Mann–Whitney *U* test (intra-host species comparison). The same test was also used to compare the numbers of mucosal-adhered canine- and chicken-derived *E. faecalis* (OD 0.5 and 1) and *E. faecium* (OD 0.5 and 1) (inter-host species comparison). The data are presented as median (range). For all analyses, we considered values of *p* < 0.05 as significant. In addition, we applied the Holm–Bonferroni correction test to deflate type 1 error and adjust the p value for our multiple comparisons. All statistical analyses were performed using the Statistical Package for Social Sciences (SPSS) software (version 22, SPSS, Inc., Chicago, IL, USA). 

## 3. Results

### 3.1. Adherence of Enterococci to Canine Duodenal Mucosa

**Figure 1:**
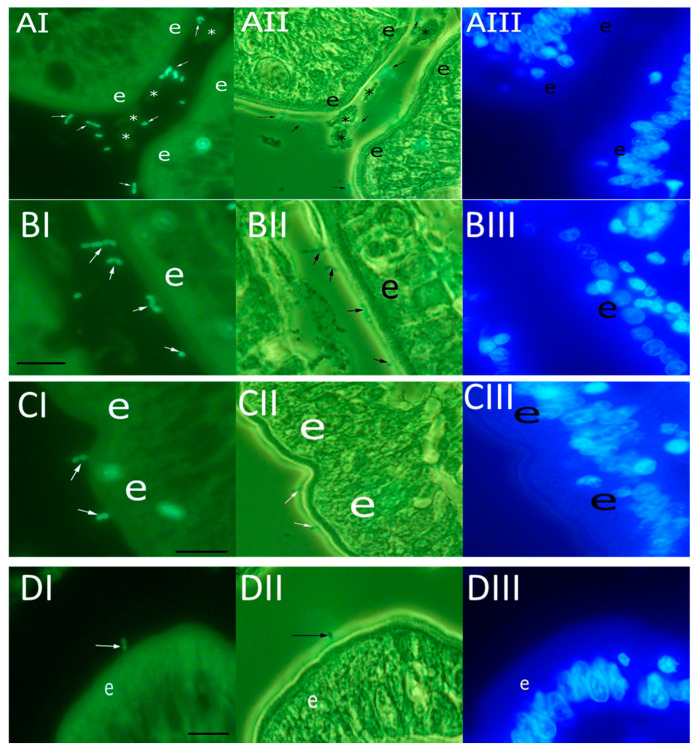
Adherence of AF488-stained *E. faecalis* and *E. faecium* to paraffin-embedded sections of the duodenal mucosa of beagle dogs. The bacterial strains shown are (**A**) canine *E. faecalis* O-67; (**B**) chicken *E. faecalis* EF368/1; (**C**) canine *E. faecium* EF397/1; and (**D**) chicken *E. faecium* EF369/9. Epifluorescence (I), epifluorescence and phase contrast combination (II), and DAPI-stained (III) images are shown. Arrows indicate bacteria adhered to the epithelium and the symbol “e” indicates the epithelial layer of the duodenal mucosa. Asterisks (*) indicate mucus. Scale bars, 50 µm.

Canine and chicken *E. faecalis* and *E. faecium* strains were labeled with AF488 and incubated on paraffin-embedded sections from the duodenum of healthy beagle dogs (Figure 1). *E. faecalis* and *E. faecium* adhered to different parts of the mucosa, including epithelium and lamina propria. Figure 1 shows the adherence of different strains to the apical surface of mucosal epithelial cells.

### 3.2. Intra-Host Species Comparison of Mucosal Adhesion of E. faecalis and E. faecium

**Figure 2:**
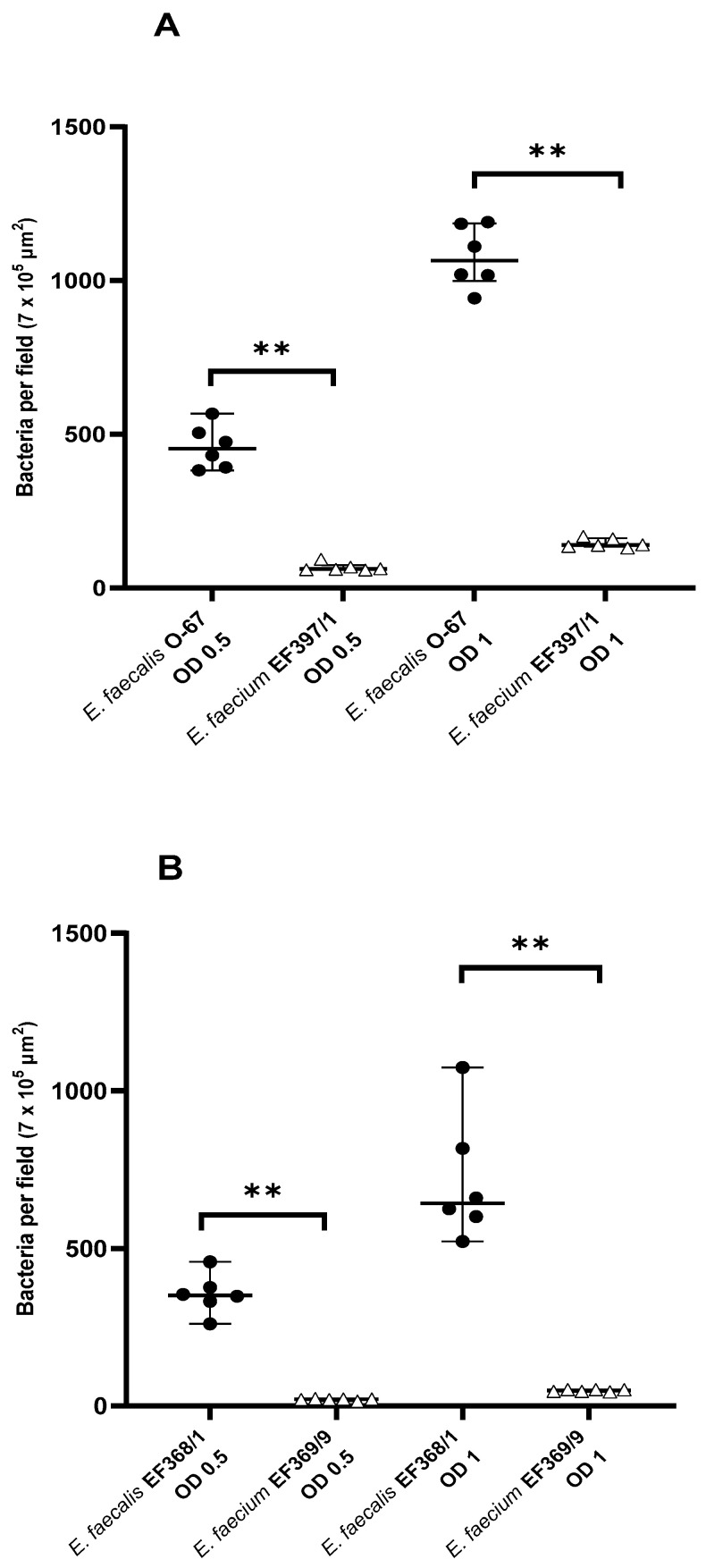
Scatter plot displaying the adherence of canine (**A**) and chicken (**B**) *E. faecalis* and *E. faecium* to the duodenal mucosa of healthy beagles. Data are expressed as the median with range. OD, optical density at 600 nm; ** *p* < 0.01.

The adhesion of canine *E. faecalis* and *E. faecium* to the duodenal mucosa of healthy beagles is shown in Figure 2A. The adherence of canine *E. faecalis* O-67 was significantly higher than that of *E. faecium* EF397/1 at both OD_600_ = 0.5 (454 (383–587) vs. 62 (59–94) bacteria per field; *p* = 0.002) and OD_600_ = 1 (1066 (943–1191) vs. 141 (131–168) bacteria per field; *p* = 0.002).

Chicken *E. faecalis* EF368/1 adhered significantly better to the duodenal mucosa of healthy beagles than *E. faecium* EF369/9 at both OD_600_ = 0.5 (351 (260–457) vs. 21 (15–23) bacteria per field; *p* = 0.002) and OD_600_ = 1 (642.5 (522–1074) vs. 49 (45–52) bacteria per field; *p* = 0.002) (Figure 2B).

### 3.3. Inter-Host Species Comparison of Mucosal Adhesion of E. faecalis and E. faecium

**Figure 3:**
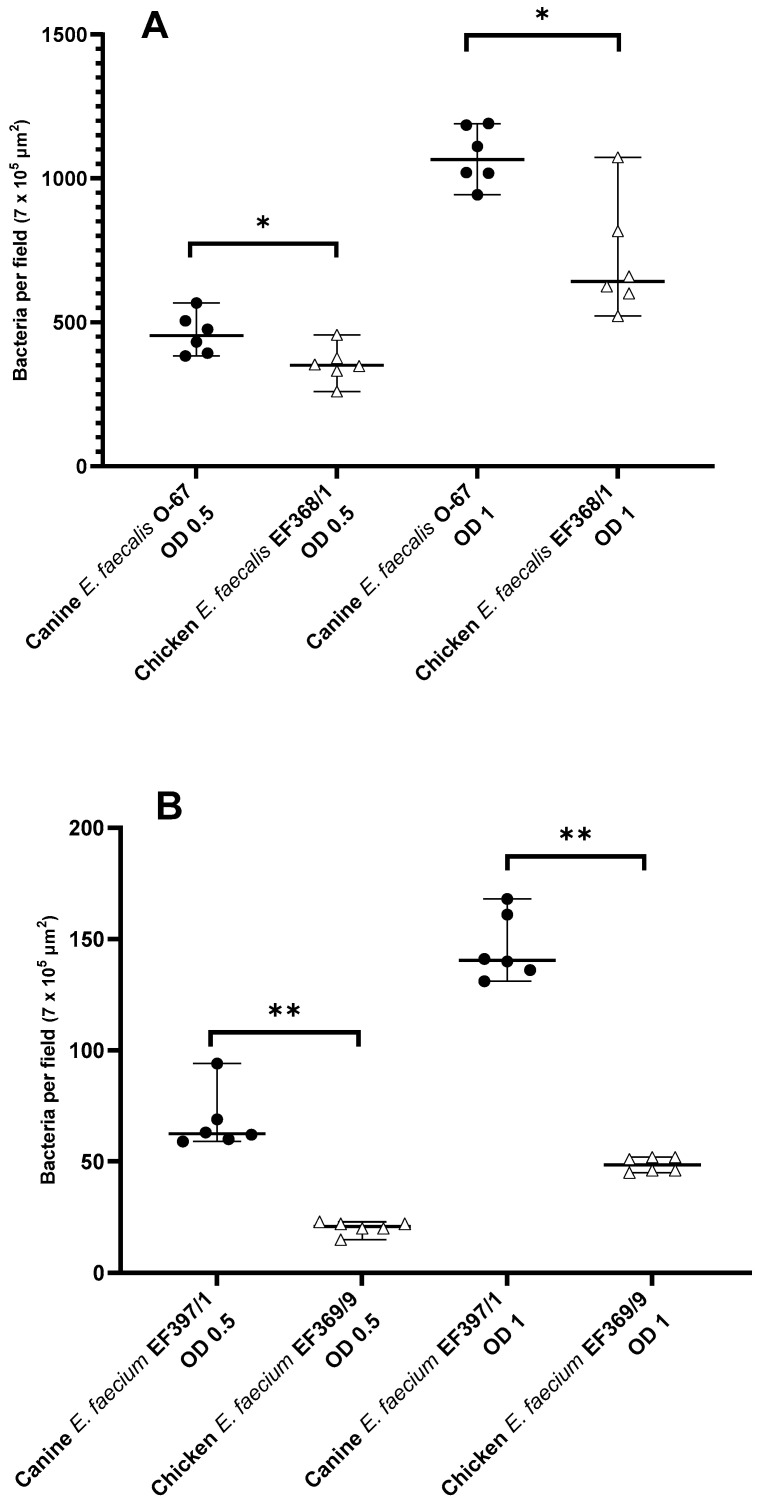
Scatter plot displaying the adherence of canine- and chicken-derived *E. faecalis* (**A**) and *E. faecium* (**B**) to the duodenal mucosa of healthy beagles. Data are expressed as the median with range. OD, optical density at 600 nm; * *p* < 0.05, ** *p* < 0.01.

The adhesion of canine *E. faecalis* and *E. faecium* to the duodenal mucosa of healthy beagles was higher when compared with that of *E. faecalis* and *E. faecium* strains of chicken origin (Figure 3). As shown in Figure 3A, canine *E. faecalis* O-67 adhered significantly better than chicken *E. faecalis* EF368/1 at both OD_600_ = 0.5 (454 (383–587) vs. 351 (260–457) bacteria per field; *p* = 0.015) and OD_600_ = 1 (1066 (943–1191) vs. 643 (522–1074) bacteria per field; *p* = 0.015). Similarly, the adhesion of canine *E. faecium* EF397/1 was significantly higher than that of chicken *E. faecium* EF369/9 at both OD_600_ = 0.5 (62 (59–94) vs. 21 (15–23) bacteria per field; *p* = 0.002) and OD_600_ = 1 (140.5 (131–168) vs. 49 (45–52) bacteria per field; *p* = 0.002) (Figure 3B).

### 3.4. Hydrophobicity of E. faecalis and E. faecium

**Figure 4:**
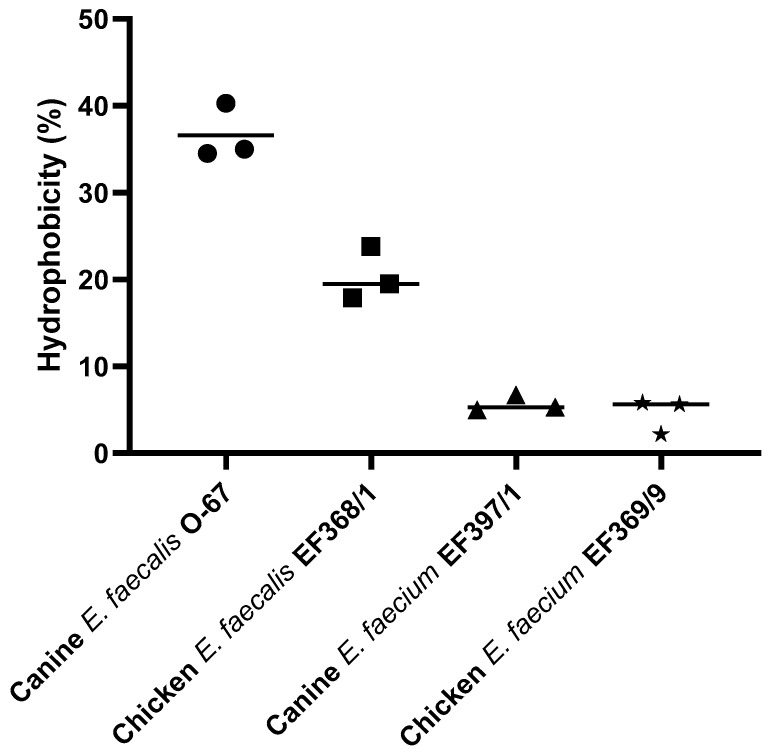
Hydrophobicity (%) of canine *E. faecalis* O-67, chicken *E. faecalis* EF368/1, canine *E. faecium* EF397/1, and chicken *E. faecium* EF369/9. Scatter plot showing data from three repetitions with their mean values indicated by black lines.

The hydrophobicity level for canine *E. faecalis* O-67 was medium (36.6%) and low for chicken *E. faecalis* EF368/1 (20.4%), canine *E. faecium* EF397/1 (5.7%), and chicken *E. faecium* EF369/9 (4.5%) (Figure 4).

## 4. Discussion

A few studies have assessed the effects of enterococci on the intestinal health of dogs and cats. Orally administered *E. faecium* has been shown to confer benefits to dogs with acute, uncomplicated diarrhea, with a better clinical outcome compared to a placebo [16]. Bybee et al. (2011) have demonstrated the probiotic potential of *E. faecium* SF68^®^ in preventing and treating diarrhea in cats housed in animal shelters. However, the strain did not have significant effects on kennel stress-associated diarrhea in dogs, which may partially be due to the low prevalence of diarrhea in this study [17]. In another study, the administration of a synbiotic containing *E. faecium* strain NCIMB 10,415 4b1707, plus the prebiotics fructooligosaccharides and gum Arabic, did not significantly alter fecal microbiota richness or diversity in dogs with or without food-responsive enteropathy (FRE) [28]. In addition, in dogs with FRE, *E. faecium* (DSM 10663/NCIMB 10415) E1707 as a single-strain treatment had no effect on the clinical activity score, histology scores, or duodenal and colonic gene expressions associated with specific T-helper lymphocyte lines [29]. Promising preliminary results were obtained with a commercially available preparation containing inactivated cells of *E. faecalis,* which reduced the number of diarrhea episodes and diarrhea severity in five out of six treated dogs with recurrent episodes of self-limiting diarrhea [18]. Further clinical studies are needed to evaluate the potential of this inactivated bacterial mixture, as no control dogs were used in this pilot study.

The ability to adhere to and colonize the intestinal mucosa of the host is important for an efficient probiotic bacterium [30]. Especially in the small intestine, where flow rates are relatively high, efficient adhesion to the intestinal mucosa is thought to be beneficial [31]. The current study is the first that investigated the adhesion capacities of canine- and chicken-derived *E. faecalis* and *E. faecium* strains to the paraffin-embedded duodenal mucosa of healthy dogs, and whether this adhesion might be related to the bacteria’s hydrophobicity. In addition, we aimed to answer the question of whether the adhesion characteristics of these bacteria are related to host specificity.

In our study, both canine- and chicken-derived *E. faecalis* strains adhered significantly better than *E. faecium* to the duodenal mucosa of healthy beagles. Rinkinen et al. (2000) also reported a relatively low level of adhesion of two *E. faecium* strains (probiotics with human origin intended for animal use) to immobilized canine intestinal mucus [20]. In another study, the *E. faecium* SF 68 and *E. faecium* M74 of human origin also showed low adherence to canine mucus compared to other lactic acid bacteria [21]. One possible reason could be the higher hydrophobicity of *E. faecalis* compared to *E. faecium.* Hydrophobicity is a physicochemical feature related to the capacity of bacteria to adhere to biological surfaces, such as the intestinal mucosa [24]. We therefore examined the affinity of canine and chicken *E. faecalis* and *E. faecium* to the hydrophobic solvent xylene. In our study, canine *E. faecalis* O-67 showed the highest hydrophobicity percentage. In contrast, chicken *E. faecalis* EF368/1 and both canine *E. faecium* EF397/1 and chicken *E. faecium* EF369/9 strains presented low levels of hydrophobicity, determined as adhesion to xylene. Interestingly, those enterococci strains that had higher hydrophobicity also adhered in higher numbers to the duodenal mucosa in our study. Higher hydrophobicity could therefore be a possible reason why *E. faecalis* strains adhered significantly better than *E. faecium* to the duodenal mucosa. Stępień-Pyśniak et al. (2019) also reported a higher level of hydrophobicity for *E. faecalis* strains than *E. faecium* strains isolated from wild birds [32]. A low level of hydrophobicity was also reported for *E. faecium* strains isolated from Brazilian cheeses and Bulgarian feta cheese (7.92% to 11.33%) [33,34]. Cell surface hydrophobicity is considered a non-specific interaction between microbial and host cells, and bacterial cells with high hydrophobicity usually present strong interactions with mucosal cells. This interaction may initially be weak and is often reversible, but may lead to more specific subsequent adhesion processes mediated by cell surface proteins and lipoteichoic acids [35,36,37].

The results of our study showed that canine *E. faecalis* and *E. faecium* adhere in higher numbers to canine duodenal mucosa compared to chicken-derived bacteria of the same bacterial species, which suggests the host-specific adhesion of these bacteria. To our knowledge, the most likely reason for the higher binding tendency of canine-derived enterococci to canine duodenal mucosa is the better host adaptation to glycan structures in the epithelium. The glycan structure varies among species, and thus, it seems that enterococci obtained from dogs are better adapted to attaching to the structures on dog epithelium [38]. The host specificity or tropism of enterococci bacteria has also been reported in other articles [9,10,39]. Dowarah et al., (2018) also showed that *Pediococcus acidilactici* FT28F isolated from piglet feces adheres heavily to pig intestinal epithelial cells without showing any adhesion towards chicken intestinal epithelial cells. In another study, a piglet-derived *Lactobacillus acidophilus* strain adhered better to intestinal epithelial cells of piglets compared to chicken intestinal epithelial cells, while the opposite was true for a chicken-derived strain [40]. Kainulainen et al. (2015) also reported that canine jejunum-derived *Lactobacillus acidophilus* LAB20 adheres better to canine colonic mucus as compared to a bacterial strain isolated from porcine colon. Interestingly, Lauková et al. (2004) compared the adhesion of *E. faecalis* and *E. faecium* strains isolated from the feces of various animals (dog, piglet, goat, horse, cattle, and rabbit) to canine, human, and porcine intestinal mucus, and did not find statistically significant differences between binding ability to any of the tested mucus types [22]. Rinkinen et al. (2003b) investigated the adhesion of various lactic acid bacteria to the intestinal mucus of humans, dogs, possums, birds, and fish. They concluded that the mucus adhesion properties of lactic acid bacteria are more dependent on the bacterial strain than on the host. However, it is worth noting that, in that study, the adherence of *E. faecium* SF 68 (human origin) was much higher to the mucus of humans and other species than to dog mucus [21].

In bacterial adhesion tests, the use of various experimental conditions (e.g., different adhesion models, bacterial strains and concentrations, buffer compositions, incubation times, and growth media) may influence the results and make inter-assay comparisons difficult [31,41]. In addition, these varying conditions may affect the results when assessing host specificity [31]. In our study, we used paraffin-embedded whole-thickness duodenal tissue sections of healthy dogs to study the adhesion properties of Alexa Fluor-labeled *E. faecalis* and *E. faecium* strains. Tissue sections give a more physiological context to the adhesion study than cultured cells, as they provide cellular organization and structures that are almost impossible to obtain using in vitro cell culture [23]. Kainulainen et al. (2015), Lauková et al. (2004) and Rinkinen et al. (2003) used a mucus model, which is based on immobilized intestinal mucus isolated from feces, jejunal chyme, or resected tissues. They radioactively labeled the bacteria, laid them over immobilized mucus, and compared the radioactivity remaining after incubation and washes to the radioactivity in the initial bacterial suspension added. Dowarah et al. (2018), in turn, used whole intestinal mucosa from pigs and chickens to study the adhesion properties of lactic acid bacteria. They incubated the bacterial suspensions on intestinal samples, after which the samples were fixed in formalin and embedded in paraffin, the slides were stained with hematoxylin and eosin and gram staining, and the attached bacteria were counted. A common disadvantage of the mucus, cell culture, and tissue section models is that they do not account for the presence of normal microbiota, which can be expected to interfere with probiotic adhesion. As summarized in Table 2, all models used in in vitro adhesion studies have their specific advantages and disadvantages; therefore, it may be advisable to assess the adhesion of potential probiotics in more than one model, each supplementing the other [41]. It is recommended to assess the adhesion of potential probiotics by using cultured cells or intestinal mucus as a prescreening method, and adhesion to whole tissue or organ culture as a second, more refined screening step [31,41].

Concerns exist over transferring antibiotic resistance genes and/or potential virulence among *Enterococcus* strains. The European Food Safety Authority (EFSA) has developed pioneering guidance for the safety assessment of one of the most common probiotics used in animal feed, *E. faecium* [42]. EFSA takes these concerns into account when assessing the safety of the probiotics containing *Enterococcus* strains and assesses each product separately [42]. Several probiotics on the market contain different strains of *E. faecium,* such as Fortiflora^®^, Nutrabio^®^, Cernivet^®^ and Cylactin^®^, or *E. faecalis*, such as Symbioflor^®^ 1. Recently, the EFSA approved using Bonvital^®^ (*Enterococcus faecium* DSM 7134) probiotic for fattening piglets in 2019 [43] and chickens in 2021 [44]. Based on EFSA guidelines, any strain of *E. faecium* demonstrating a resistance to ampicillin greater than 2 mg/L or possessing any of the three virulence marker genes (IS*16*, *Esp*, and *Hyl_efm_*) should not be used as a feed additive [42]. Similar measures are also applied for *E. faecalis* strains proposed for use in the food or pharmaceutical industries.

In our current study, we used archived formalin-fixed samples from healthy beagles, deparaffinized by xylene and ethanol, to investigate the adhesion properties of *E. faecalis* and *E. faecium* to the duodenal mucosal. Carnoy’s fixative can reportedly be superior to formalin in preserving the mucus layer [45], but we used formalin-fixed and xylene- and ethanol-deparaffinized samples for the quantification of bacteria in a previous study, and demonstrated a largely intact mucus layer in the colonic mucosa of dogs [46]. Other studies have also utilized the same methods for quantifying bacteria in the intestinal mucosa of dogs and cats [47,48].

Despite the increasing number of probiotic products marketed for dogs, there are surprisingly few studies on one of the most important properties of probiotic bacteria, i.e., their ability to attach to the intestinal mucosa. Studies concerning metabolic mechanisms of bacterial crosstalk with canine intestinal mucosa are completely missing. All evidence concerning probiotic adhesion and its possible effects on the intestinal mucosa is based on analogies from other species. Our study is an attempt to overcome these shortcoming by applying, for the first time, a recently published new method [23] that allows for studying bacterial attachment to paraffin-embedded intestinal tissue. The method allows for studying bacterial attachment in a more physiological context by providing organizations and structures comparable to immobilized mucus [5,20,21] or isolated intestinal epithelial cells [5]. Considering the tradition in classical pathology of archiving paraffin-embedded biopsy material, the method also allows for bringing these vast resources to new use in probiotic research. Furthermore, archived paraffin-embedded intestinal tissue samples can be used to avoid the unnecessary use of animals for experiments, thereby contributing to the principles of their replacement, reduction, and refinement [49]. As a first step in studying the effects of probiotic bacteria on canine intestinal mucosa, we investigated whether selected bacteria do adhere before using more advanced methods to study the intestinal barrier’s fortifying and anti-inflammatory effects, or the metabolic mechanisms of crosstalk.

The main limitation of our study is its small sample size. Basically, this research was more an exploratory than a confirmatory study, and the observed results could be due to chance. In the current study, result variability has been reduced by analyzing the mean of three runs, which is a good estimate of the attached bacteria in a sample. Due to the small sample size and non-normally distributed data, we used the non-parametric Mann–Whitney U test. However, we also analyzed the data with parametric testing (Independent Samples *T*-test), and the results were similarly significant, which indicates their robustness. In addition, we applied the Holm–Bonferroni correction test to deflate type 1 error, and adjusted the *p*-value for multiple comparisons. Even after adjustment, the *p*-value remained significant, which proves the accuracy of our results.

## 5. Conclusions

The results of this study showed that canine *E. faecalis* and *E. faecium* adhere in higher numbers to canine duodenal mucosa compared to chicken-derived bacteria of the same species. In addition, both canine- and chicken-derived *E. faecalis* strains adhered significantly better than *E. faecium* to the duodenal mucosa of healthy beagles. The adhesion properties seem to be associated with bacterial hydrophobicity. In this study, we used, for the first time, paraffin-embedded dog intestinal tissue sections to investigate the adhesion properties of Alexa Fluor-labeled *E. faecalis* and *E. faecium*. Our results suggest that both the bacterial strain and the host may influence the mucosal adhesion properties of *E. faecalis* and *E. faecium*. Host gut-derived bacteria might be preferable as probiotics, as they have adapted to the gastrointestinal environment of the host and can proliferate and express biological activity in a more competitive way compared to microbes from other host species.

## Figures and Tables

**Table 1:** List of bacterial strains used in this study.

	Strain	Origin
*Enterococcus faecalis*	O-67	Dog feces; isolated at the Central Laboratory of the Department of Equine and Small Animal Medicine, University of Helsinki, Helsinki, Finland
*Enterococcus faecalis*	EF368/1	Chicken cecum; isolated at the Microbiome Laboratory, Orion Corporation, Orion Pharma, R&D, Turku, Finland.
*Enterococcus faecium*	EF397/1	Dog feces; isolated at the Microbiome Laboratory, Orion Corporation, Orion Pharma, R&D, Turku, Finland.
*Enterococcus faecium*	EF369/9	Chicken cecum; isolated at the Microbiome Laboratory, Orion Corporation, Orion Pharma, R&D, Turku, Finland.

**Table 2:** Advantages and disadvantages of various types of in vitro bacterial adhesion models (Modified from Van Tassell and Miller, 2011) [41].

Adhesion Model	Description	Advantages	Disadvantages
Cell culture	Polar monolayer of enterocytes resembling intestinal tissue	Provides conditions more similar to in vivo environment	Derived from cancer cells, could differ from healthy tissue. Not representative of cell-type ratios in mucosal epithelial tissues
Caco-2/HT29	Caco-2 and HT29 carcinoma cell lines	Simple, well-established in literature	Does not account for mucus presence
HT29-MTX/FU	HT29 culture treated with methotrexate or fluoruracil to secrete various types of mucus	Accounts for presence of mucus	May not represent appropriate *MUC* gene expression
Co-cultures	Mixed culture of secreting and mucus-secreting cells	Better represents cell-type ratio of mucosal epithelial tissues	Little literature on use in adhesion studies
Immobilized mucus	Mucus preparations immobilized, usually in microtitre wells	Fast, isolates mucus-microbe interactions from other in vivo conditions	Difficult to separate mucus, specifically from hydrophobic interactions
Whole tissue	Whole, intact, or excised tissue	Provides in vitro conditions most similar to in vivo environment	Costly, difficult to obtain
Resected tissue	Fragments of tissue excised from host	Mucus, epithelial tissue, and commensal organisms accounted for in model	Only small fragments at a time available from living hosts
Organ culture	Whole organs maintained in vitro	Better maintains the architecture of the tissue	Prohibitively expensive, may not function in same manner as in vivo

## Data Availability

Not applicable.
